# Regional Differences in Various Risk Factors for Postpartum Depression: Applying Mixed Models to the PRAMS Dataset

**DOI:** 10.3389/fgwh.2021.726422

**Published:** 2021-10-29

**Authors:** Janace J. Gifford, Jenna R. Pluchino, Rebecca Della Valle, Jaclyn M. Schwarz

**Affiliations:** Department of Psychology, University of Delaware, Newark, DE, United States

**Keywords:** postpartum depression (PPD), education, health care, pregnancy, risk factors, symptom severity

## Abstract

**Purpose:** The purpose of this study was to assess the association between various risk factors with postpartum depression severity using a large dataset that included variables such as previous mental health status, social factors, societal factors, health care access, and other state-wide or region-specific variables.

**Methods:** We obtained the most recently available (2016–2017) dataset from the Pregnancy Risk Assessment Monitoring System (PRAMS), which is a dataset compiled by the Centers for Disease Control (CDC) that collects state-specific, population-based data on maternal attitudes and experiences before, during, and shortly after pregnancy from over 73,000 women in 39 states. We utilized a hierarchical linear model to analyze the data across various levels, with a symptom severity scale (0–8) as the dependent variable.

**Results:** Of the 21 variables included in the final model, nine variables were statistically significant predictors of symptom severity. Statistically significant predictors of increased postpartum depression symptom severity included previous depression diagnosis and depression symptoms during pregnancy, baby not residing with mother, unintentional pregnancy, women with less than a high school degree and more than a college degree, Women Infants Children (WIC) enrollment, and married women. In contrast to these other factors, attendance at a postpartum follow up appointment was associated with significantly increased symptom severity. Age revealed an inverted curve in predicting postpartum symptom severity.

**Conclusions:** There was no significant difference in symptom severity scores across the 39 participating states. Most notably, postpartum depression symptom severity was associated with previous depression diagnosis and previous symptom severity, but our results also reveal novel social and education factors that contribute to the support and well-being of the mother and child.

## Introduction

According to the Centers for Disease Control and Prevention (CDC), about 1 in 9 women experience symptoms of postpartum depression (PPD) after the birth of a child (1). As such, postpartum depression should be considered an important area of study in the basic biomedical and clinical research communities. However, the classification of PPD as a mental health disorder that is unique from Major Depressive Disorder is controversial; and thus there has been very little research investigating its unique etiology or risk factors. Literature on postpartum depression shows a correlation between stress and the likelihood of being diagnosed, although the source of that stress can vary. Previous studies have shown IPV (intimate partner violence) (2–9), being unmarried, unwanted pregnancy (4, 6, 7, 9–11), and prenatal anxiety or depression to be significant risk factors for PPD. In contrast, factors like social support (12, 13), being older (4, 6, 8, 9, 11, 13–17) achieving higher levels of education (4, 6, 11, 13, 15, 17), and a higher income (4, 6, 8, 9, 14) have been shown to be protective factors. Surprisingly, only 0.2% of basic biomedical research in the field of depression mentions postpartum mood disorders.

Similarly, the current diagnostic systems used by the American Psychiatric Association and the World Health Organization *do not* consider PPD a distinct mental health disorder, but rather part of a subtype of major depressive disorder (MDD). In the DSM-5, major depressive disorder includes a subset classified as “a major depressive episode with peripartum onset” which is defined as onset of symptoms during pregnancy and/or the first 4 weeks postpartum, therefore including both peripartum and postpartum depression (18). Notably, this is an improvement from the DSM-IV, which did not include the onset of depression during pregnancy. In the United States, however, mothers are not screened for PPD until their first and only 6-week postpartum follow-up appointment with their obstetrician, which presents a barrier to timely care and prevention, but also our understanding of the factors that predict the risk and severity of PPD is relatively limited. The importance of studying state level variables in predicting PPD symptom severity stems from the accessibility of healthcare providers (measured by providers per capita) (19), in addition to the Medicaid threshold for pregnant women and adult/parent caretaker in each state (20). With this research project, we hypothesized that social and societal factors, as well as access to medical care and insurance may contribute to the severity of postpartum depression symptoms in women across the US in a region specific manner. To examine this, we used the most recently available dataset of the Pregnancy Risk Assessment Monitoring System, or PRAMS, from 2016 to 2017. This dataset is compiled by the CDC from women via a questionnaire sent by mail 3–4 months after a mother's delivery date. The data from PRAMS is used to document issues impacting adverse birth outcomes, but also includes questions about postpartum mental health. The dataset includes responses from over 73,000 women in 39 states.

## Materials and Methods

A hierarchical linear model was utilized to analyze postpartum depression severity and its association with variables collected from the PRAMS dataset. This dataset is compiled from women *via* a questionnaire sent by mail 3–4 months after a mother's delivery date. The data from PRAMS is used to document variables impacting adverse birth outcomes, but also includes questions about postpartum mental health. The dataset includes responses from over 73,000 women in 39 states. The dataset used was reviewed and deemed exempt from IRB at the University of Delaware as all personal information had been de-identified prior to receiving the dataset from the CDC.

We also sought to determine how variables at the state level might affect PPD risk factors for individual women. We analyzed the healthcare provider data by categorizing the providers based on specialties to determine how many would be accessible specifically to pregnant women. Specialties included psychiatrist, psychologist, family/marriage counselors, and mental health counselors. Medicaid cutoff data was specifically organized into thresholds for pregnant women and thresholds for adult/parent caretaker. These variables were added to the dataset using data from reports by The Henry J. Kaiser Family Foundation and U.S. Department of Health and Human Services, Health Resources and Services Administration, and the National Center for Health Workforce Analysis.

Variables at the lower level of our analysis include a variety of demographic variables as well as variables related to health care utilization, prior depression, and stress. These variables included mother's age (M = 29.46, SD = 5.79), mother's race (57.8% white, 19.1% black, 23.1% other) household income (M = 6.62, SD = 4.542), whether the baby lived with the mother at the time of survey (95.3% responded yes), prior depression diagnosis (13.9% has prior diagnosis), infant mortality (0.5% mortality), pregnancy intendedness, marital status (40.1% unmarried, 59.9% married), attendance at a postpartum checkup (87.9% attended), mother's education level (M = college or more), whether the mother reported smoking (88.9% reported no), number of previous births (M = 1.01, SD = 1.061), paternity acknowledgment (26.7% yes, 17.6% no, 55.7% no data), length of baby's hospital stay (M = 0.649 days, SD = 0.993) number of dependents (M = 2.97,SD = 1.416), prenatal care quality, whether the mother reported breastfeeding (78.8% did breastfeed, 19.1% did not), and WIC enrollment (39.5% enrolled). Special Supplemental Nutrition Program or Women, Infants, Children (WIC) is a government supported program in the United States that aims to protect the health of lower income women, infants and children up to age 5 who may be at nutritional risk. This program provides nutritious foods, nutrition education and supplement diets as well as access to other health and social services (21).

The dependent variable was PPD symptom severity, which was determined by taking two of the PRAMS questions about depression and anhedonia and combining them to generate a single postpartum depression symptom severity score. These two questions were, “Since your new baby was born, how often have you felt down, depressed, or hopeless?” and “Since your new baby was born, how often have you had little interest or little pleasure in doing things you usually enjoyed?” Each utilized a 5 point Likert scale where 0 is never and 4 is always, thus combining the score from these two questions resulted in a symptom severity scale ranging from 0 to 8, where 0 is no symptoms at all and 8 is the most severe symptoms. The results from the first hierarchical linear model including state level and lower level variables reflected no significant effect on postpartum symptom severity, therefore state level variables were removed from the final model. This final hierarchical linear model included 21 predictor variables listed in Table 1. The estimates (regression coefficients) at the individual level for each of these variables are listed in Table 1 in order of overall effect size. Significant estimates (with an α = 0.0005) of factors is indicated by an asterisks, and the *p*-value is listed.

**Table 1:** Predictors observed. Listed are the 22 variables observed of which 9 (indicated with *) were found to be significant predictors of symptom severity.

**Variable**		**Estimate**	***F***	**Sig**.
Depression symptoms during pregnancy	*	1.579403	1081.669	0.000
Does baby live with mother?	*	−0.955017	57.320	0.000
Depression diagnosis prior to pregnancy	*	0.580786	145.453	0.000
Infant mortality		0.422144	0.428	0.513
Pregnancy intendedness	*	−0.297919	87.694	0.000
Marital status	*	0.195915	7.012	0.008
Postpartum checkup	*	−0.130486	9.101	0.003
Mother's education	*	0.080590	21.285	0.000
WIC enrollment	*	0.089245	6.787	0.009
Smoking		0.069559	2.266	0.132
Breastfeeding		0.041069	1.310	0.252
Rural or urban?		0.043996	1.692	0.193
Number of previous births		0.020780	1.173	0.279
Paternity acknowledgment		0.028463	0.632	0.427
Length of baby's hospital stay		0.015809	1.089	0.297
Number of dependents		0.015676	1.510	0.219
Type of insurance		0.010998	0.549	0.459
Mother's age	*	−0.009806	9.386	0.002
Prenatal care quality		−0.009029	0.377	0.539
Mother's race		0.007702	1.497	0.221
Household income		−0.003741	0.348	0.555

## Results

**Figure 1:**
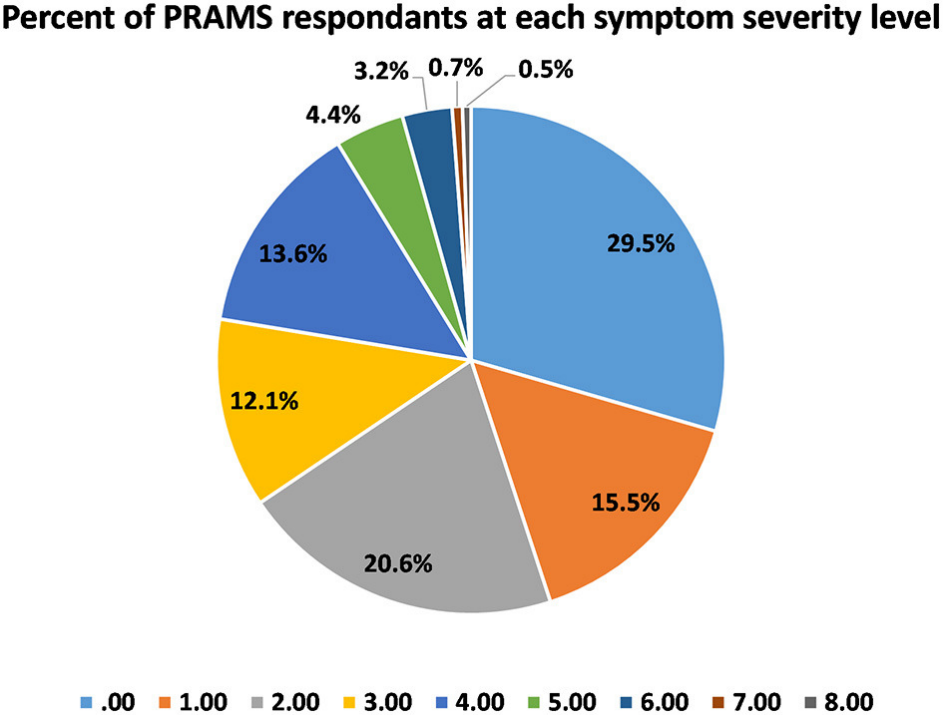
Distribution of symptom severity scores across PRAMS respondents.

The frequency distribution for our symptom severity scale revealed that 29.5% of women scored a 0, while 0.5% scored an 8 (Figure 1). To put this into perspective, 1 in 200 new mothers were experiencing severe postpartum depression symptoms at the time of the survey.

At the upper or state level, differences in symptom severity were accounted for by only 0.56% of the variance, and contrary to our hypothesis, none of the state-level variables (including the medicaid cut off rates and the number of mental health care providers per capita) improved our model, and so these variables were not included in the final or subsequent analyses.

**Figure 2:**
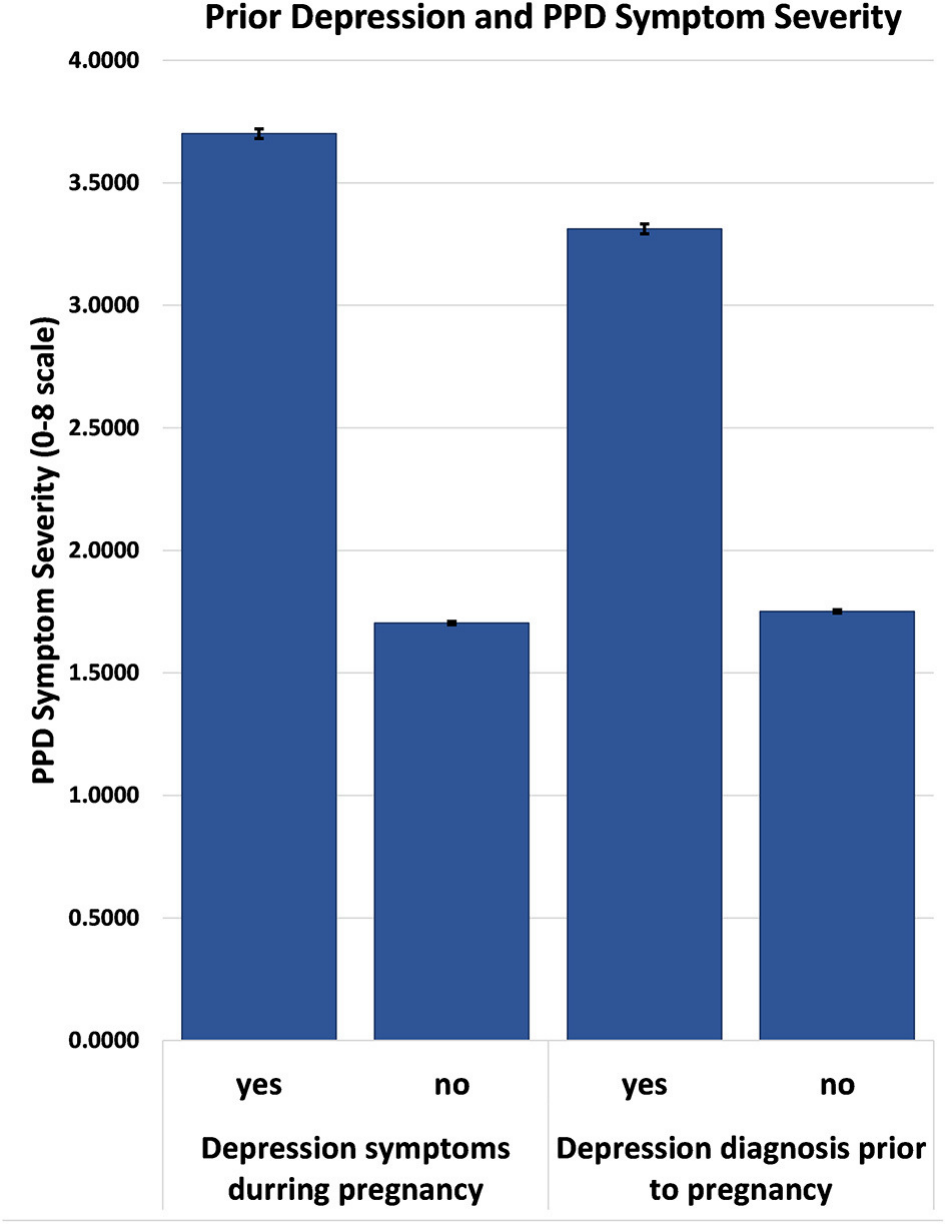
Postpartum symptom severity based on response to previous depression diagnosis and depressive symptoms during pregnancy.

At the lower level of our modeling, having depression symptoms *during* pregnancy was associated with a 1.58 point increase in the symptom severity of postpartum depression, the largest effect size and greatest predictor in our model. Similarly, having a depression diagnosis prior to pregnancy was associated with a 0.58 point increase in symptom severity (Figure 2).

**Figure 3:**
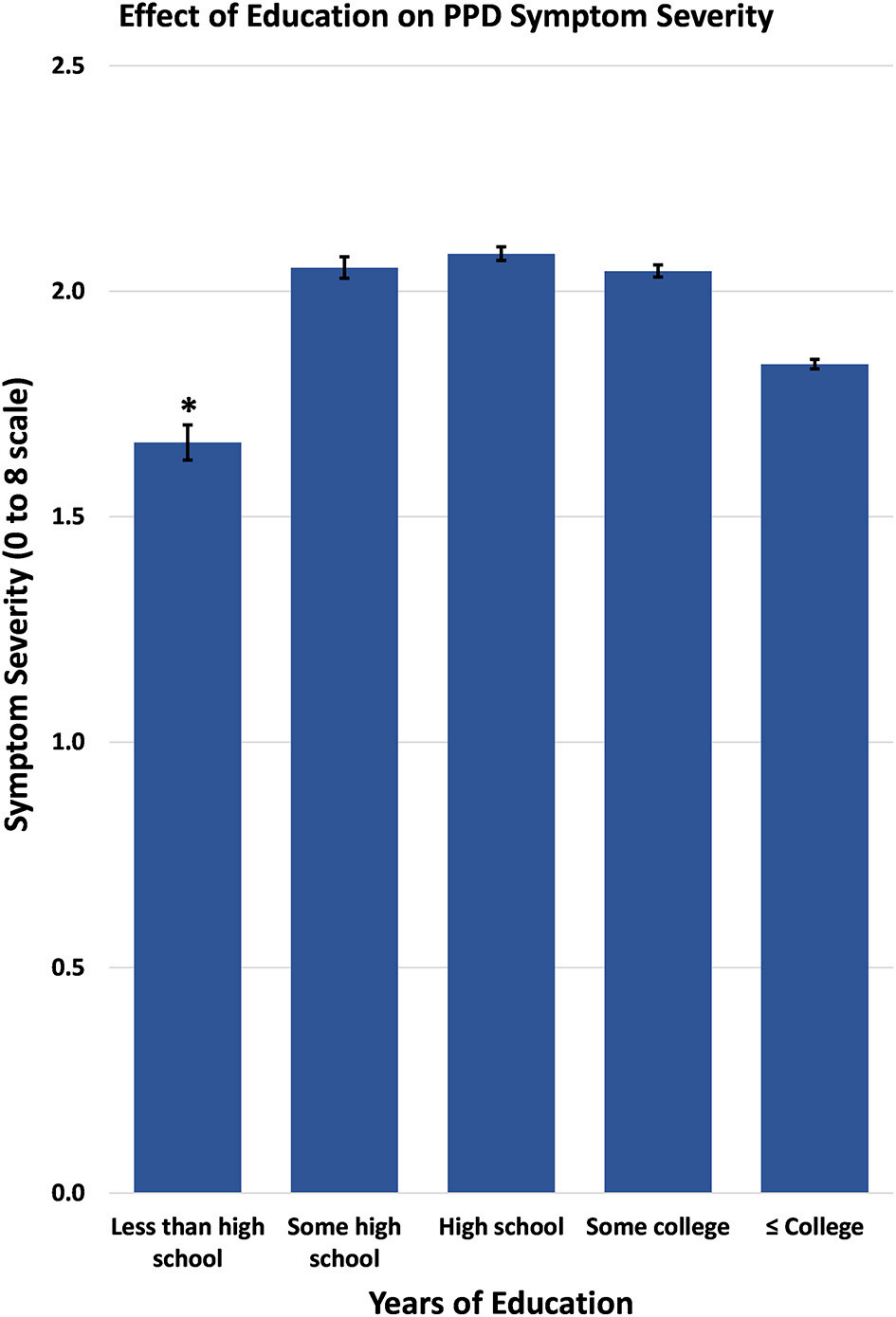
Postpartum symptom severity by education level as reported by PRAMS respondents.

Other social and personal factors analyzed at the lower level of our model also accounted for the difference in postpartum symptom severity. Specifically, if the infant is not residing with the mother, it was associated with a 0.96 point increase in symptom severity. Moreover, reporting the pregnancy as “unwanted” or having been “wanted later” was associated with a 0.30 point increase in postpartum depression symptom severity compared to women who reported their pregnancy as “wanted now” or had “desired having it sooner.” Being married was associated with a 0.20 increase in symptom severity, as compared to unmarried women. Attending postpartum checkups was also associated with a 0.13 point increase in symptom severity. Participation in the Women, Infant's, and Children's Nutritional Program (WIC), which is available to mothers who qualify based on low income, was associated with a 0.09 point increase in postpartum depression severity. Maternal education was also a significant predictor (0.08), with women at the highest and the lowest levels of education exhibiting fewer symptoms of postpartum depression (Figure 3).

In contrast to the other factors analyzed, there was a *negative* association between age and symptom severity with a decrease of 0.0098 points. Interestingly, in this model, race and household income were not predictors of postpartum depression symptom severity, regardless of previous literature that has identified such an association. A full list of lower level variables and their effect sizes can be found in Table 1.

## Discussion

The current analysis of the PRAMS dataset considered the various state-specific, social, socioeconomic, and previous depression as risk factors for postpartum depression severity in the United States. This analysis is unique in the number and variety of important variables considered in the risk of postpartum depression, as well as the use of the most recently available PRAMS data set. Our analysis revealed that only 29.5% of women surveyed experienced no symptoms of PPD or changes in postpartum mood. Thus, our analysis revealed that 70.5% of women surveyed experienced some change in mood during the postpartum period. First these numbers are similar to other reports that indicate ~60% of women experience some change in mood postpartum, 20% of whom go on to be diagnosed with PPD (22, 23). In the current analysis, 21.9% of women experienced moderate (score = 4/8) to severe (score = 8/8) levels of PPD symptoms, which is a large number of the women surveyed, highlighting the importance of research examining the causes of PPD. Similar to previous findings, the strongest predictor of postpartum symptom severity occurred if a mother had ever received a prior depression diagnosis or if she had experienced depression symptoms during pregnancy. These two factors predicted postpartum depression severity to a similar degree (*p* < 0.001). The latter highlights the importance of considering and including symptoms during pregnancy in the diagnosis of postpartum depression. This finding also suggests that the general neurobiological or physiological factors that increase one's risk of depression in general, also contributes to the subsequent risk of postpartum depression after birth. These data are in line with and replicate previous findings of the same nature, indicating that previous episodes of depression, a depression diagnosis, or depressive symptom particularly during the third trimester significantly increase the risk of postpartum depression (4–6, 8–10, 14, 24). Moreover, these data support the current classification of PPD as a subset or type of Major Depressive Disorder.

The present study also observed a number of personal and social characteristics as potential risk factors for postpartum depression. Particularly, education level was associated with less severe postpartum symptoms at the lowest and highest levels of education. In prior studies which tested for educational differences contributing to PPD, it was found that there was a consistent negative association between education and PPD symptom prevalence indicating that greater education is associated with a decrease in PPD symptom severity (6, 11, 17, 23, 25). This finding of lower levels of education being associated with decrease symptom severity was surprising but may be due to the distribution of PRAMS respondents−3% of respondents had less than a high school degree while 33.7% had the highest levels of education (college or more). The lack of participants at the lowest level of education may not accurately represent the prevalence of PPD symptom severity among the lowest education level. The current analysis revealed that married women (59.9% of women surveyed) were more likely to report postpartum symptoms than unmarried women (40% of women surveyed), a finding that is consistent with a previous finding using an earlier PRAMS dataset from 2009 to 2011 in which 56.2% of women reported being unmarried and 43.8% of women reported being married (10, 17). These other investigations also indicate that increased relationship stress is a predictor of postpartum depression severity, while decreased relationship stress is a protective factor in the risk of postpartum depression (26) and thus should be considered in the interpretation of these current results as well.

**Figure 4:**
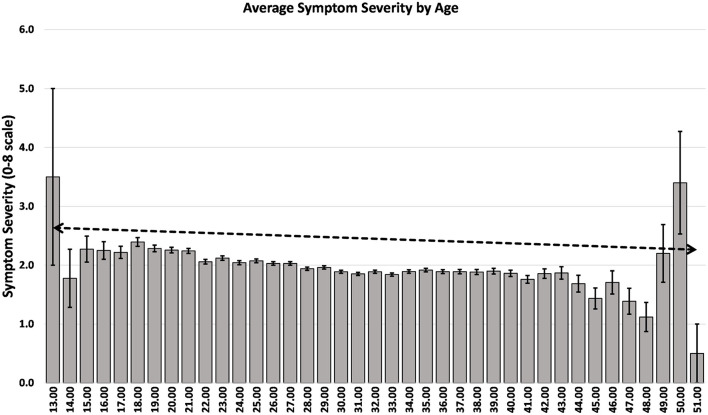
Average symptom severity of PRAMS respondents across age of respondent.

In the present study we observed that age was associated with postpartum symptom severity, younger women reported more postpartum symptoms than older women with a notable decrease in symptom severity with every 10 years of age which replicates similar previous findings (Figure 4) (2, 4, 6, 9, 13, 15, 17). This finding suggests age may be an important risk factor for postpartum depression and younger mothers in particular should be screened for PPD.

Consistent with previous literature, WIC enrollment was associated with a slight increase in symptom severity (3, 8, 16). Previous work has indicated women enrolled in WIC or women who are WIC eligible are more likely to experience PPD compared to non-eligible women and suggests that WIC enrollment itself may not be a risk factor for PPD but rather WIC eligibility or experiencing poverty itself may lead to increased PPD risk (7). Moreover, the current results found that symptom severity was elevated when the infant did not reside with the mother and when pregnancy was unintended which replicated previous findings (3, 4, 6, 7, 10, 27), and may relate to the inability of the mother to effectively bond with her new infant in these situations.

Unexpectedly, the present analysis revealed that women who attended their 6 week postpartum appointment reported increased postpartum severity. Although this was a surprising finding, it may be that women who attended and were assessed for PPD at this check in were more likely to recognize their symptoms than women who did not attend their 6 week check in. It is important to note that in the US it is common that women receive only one check in appointment at 6 weeks postpartum. Although the 6 week appointment is beneficial to assess for PPD symptoms, this single 6 week check-in and lack of structured interviews with women postpartum is limiting for research purposes and overall potentially detrimental to women's mental health postpartum.

We predicted that taking into account state level variables such as the mental health care providers per capita and Medicaid cut off rates would reveal regional differences in postpartum depression risk factors. Analysis of the state level variables did not reveal a significant correlation between postpartum risk factors and certain areas of the country. It is possible that these effects were due to the fact that stressors which cause postpartum symptoms such as education level, SES, and marital status are not unique to one particular location. Regardless of location, women can experience the same type of hardships which in turn can exacerbate postpartum symptoms.

It is recognized that other factors not observed in the present analysis may play a role in the development of PPD and may include other etiological or biological factors, levels of social support and individual psychological and coping mechanisms. Future datasets should examine women during pregnancy and postpartum for a more comprehensive understanding of the development and risk factors for postpartum depression. Overall, the results of the present study support the future implementation of a more integrated approach to assessing women at risk for PPD by screening women throughout pregnancy for risk factors to maximize intervention success.

## Data Availability Statement

Publicly available datasets were analyzed in this study. PRAMS, the Pregnancy Risk Assessment Monitoring System, is a surveillance project of the Centers for Disease Control and Prevention (CDC) and state health departments. It is available to any researchers by submitting a proposal to the CDC.

## Author Contributions

RD and JS: study conception and design. JP, RD, JG, and JS: analysis and interpretation of results. JG, JP, and JS: draft manuscript preparation.

## Funding

This work was funded by the National Institutes of Health grant R21MH122862 to JS and a University of Delaware Summer Scholars Research Fellowship to JP. All authors contributed to the article and approved the submitted version.

## Conflict of Interest

The authors declare that the research was conducted in the absence of any commercial or financial relationships that could be construed as a potential conflict of interest.

## Publisher's Note

All claims expressed in this article are solely those of the authors and do not necessarily represent those of their affiliated organizations, or those of the publisher, the editors and the reviewers. Any product that may be evaluated in this article, or claim that may be made by its manufacturer, is not guaranteed or endorsed by the publisher.
